# Association of anxiety and depression with cognitive challenges in children in Bangladesh

**DOI:** 10.1371/journal.pone.0343489

**Published:** 2026-02-20

**Authors:** M. Iftakhar Alam, Mohaimen Mansur

**Affiliations:** Institute of Statistical Research and Training, University of Dhaka, Dhaka, Bangladesh; Chittagong Medical College, BANGLADESH

## Abstract

This study primarily investigated the association of anxiety or depression (AD) with communication, learning, remembering, or concentrating (CLRC) difficulties in children aged 5-17 years in Bangladesh. Data were taken from the 2019 Bangladesh Multiple Indicator Cluster Survey (MICS). Random intercept binary logistic regression model was used to assess the association between CLRC and AD, after controlling sex, age, division, maternal education, maternal functional difficulty, and wealth index. Children with AD difficulties had significantly higher odds of CLRC (OR = 7.82; 95% CI: 6.29–9.73). Males were 30% more likely to have CLRC than females (OR = 1.30; 95% CI: 1.12–1.51). Compared with children living in Barishal-the reference administrative region-children residing in several other divisions had significantly lower odds of experiencing CLRC. Children of mothers with secondary education had reduced odds of CLRC (OR = 0.80), while those whose mothers had functional difficulties had higher odds (OR = 3.00). To conclude, AD difficulties is strongly associated with CLRC, alongside other factors like sex, geographic location, maternal education, and wealth. Addressing these factors is crucial for reducing CLRC difficulties in children.

## 1 Introduction

Childhood is a critical developmental period where emotional and cognitive foundations are established, significantly influencing future mental and physical well-being. During the ages of 5 to 17, children undergo rapid psychological, social, and cognitive development, which can be affected by various factors, including mental health issues like depression and anxiety. Studies indicate that early-life mental health conditions can interfere with cognitive processes such as communication, learning, remembering, and concentrating, potentially hindering academic performance and social interactions [1]. The interaction between mental health and cognitive functioning in children is multifaceted, involving both biological and environmental factors [2]. Luby et al. [3] indicated that children with depressive symptoms are more likely to experience cognitive impairments, particularly in areas such as working memory, verbal learning, and attention. Kaufman et al. [4] found that early-onset depression can negatively impact academic achievement, as children with depression frequently struggle with tasks requiring sustained concentration and problem-solving. The cognitive deficits associated with depression are especially evident in school-aged children (7-12 years), where learning and retention of new information are critical.

Anxiety disorders are among the most common mental health conditions in children and adolescents, often emerging between the ages of 5 and 17 [5]. Anxiety typically manifests as excessive worry, fear, and hyper-vigilance, which can disrupt cognitive processes such as attention, working memory, and executive functioning. Anxiety increases cognitive load, reducing the brain’s ability to focus on tasks and maintain attention [6]. Children with anxiety often struggle in academic settings, where tasks demand sustained concentration and the ability to shift focus between different activities. Anxious children often exhibit impairments in working memory, which is crucial for learning, problem-solving, and information retention. These cognitive challenges are most pronounced during tasks that involve multiple steps or require the child to hold and manipulate information over time.

Cognitive abilities like working memory and attention are critical for academic success, particularly in the early stages of formal education [7]. Children who struggle with these cognitive functions often face challenges in literacy and numeracy, which can impact their long-term academic trajectories [8]. Learning difficulties in young children with depression or anxiety are frequently linked to deficits in executive functions, including planning, organization, and flexible thinking [9]. These children may have trouble understanding instructions, organizing their thoughts, or remembering important information, all of which are essential for effective learning. Cognitive difficulties related to attention, memory, and learning can also hinder social interactions, as children with these challenges may struggle to engage in conversations or remember social cues, leading to social withdrawal or frustration [10].

Previous studies have consistently documented a relationship between anxiety and depression and impaired cognitive functioning in children and adolescents. Evidence from clinical and school-based studies indicates that depressive symptoms are associated with deficits in attention, working memory, executive functioning, and academic performance [3,4,8]. Similarly, anxiety disorders have been linked to reduced attentional control and working memory capacity, which can adversely affect learning and concentration [6,7]. Population-based studies from high-income settings also report higher prevalence of learning and concentration difficulties among children experiencing emotional or internalizing disorders [5].

The Child Functioning Module (CFM) has been developed by the Washington Group on Disability Statistics and UNICEF to obtain reliable data [11,12]. Experts in the area have extensively reviewed the module. Moreover, it has been tested in several countries to validate the questions being asked and ascertain the respondents’ cultural understanding [13–15], and it was finalized in 2016. The CFM has been utilized for the first time in Bangladesh in MICS 2019 and this paper utilized the data of 5-17 years children from this survey.

Although the association between anxiety, depression, and cognitive functioning in children has been widely reported, most existing evidence comes from clinical samples or studies conducted in high-income countries. There is a notable lack of population-based evidence from low- and middle-income countries. Moreover, few studies have examined this association using standardized functional disability frameworks, such as the UNICEF/Washington Group CFM, which allows for internationally comparable results. In the context of Bangladesh, nationally representative evidence linking anxiety- or depression-related functional difficulties to cognitive challenges among children remains scarce, despite the growing policy relevance of child mental health and disability monitoring.

The primary objective of this study was to examine the association between functional difficulties in the forms of anxiety or depression (AD) and cognitive challenges-defined as difficulties in communication, learning, remembering, and concentrating (CLRC)-among children aged 5-17 years in Bangladesh. Using nationally representative MICS 2019 data, we assessed whether children with AD-related functional difficulties have a higher likelihood of experiencing CLRC, while adjusting for key demographic, geographic, socioeconomic, and maternal characteristics. A secondary objective was to assess the associations of these covariates as well with CLRC.

## 2 Materials and methods

### Data

The data for this study came from the 2019 Multiple Indicator Cluster Survey (MICS), conducted by the Bangladesh Bureau of Statistics in collaboration with UNICEF Bangladesh [16]. The survey aimed to provide nationally representative estimates on various indicators related to the well-being of children and women across Bangladesh’s eight divisions and sixty-four districts. A two-stage stratified cluster sampling method was employed. The districts served as the primary sampling strata, and households were selected through a two-step process. First, enumeration areas were systematically chosen with a probability proportional to their size. Then, within each selected area, 20 households were systematically sampled following a household listing. The dataset used in this analysis, which focuses on children aged 5–17, was released in March 2020 and includes a sample of 66,705 children.

### Ethical approval

The survey was conducted by the Bangladesh Bureau of Statistics, which obtained ethical clearance prior to its implementation. As this study utilized secondary data, additional ethical approval was not required for the authors. The dataset is available at the following link: https://mics.unicef.org/.

### Outcome variable

The CFM component for 5-17 years children covered 13 core domains of functioning, which included seeing, hearing, walking, self-care, communication, learning, remembering, concentrating, accepting change, controlling behavior, making friends, anxiety and depression. Most of the module questions included response categories: ‘no difficulty’, ‘some difficulty’, ‘a lot of difficulty’ and ‘cannot do it at all’. If responded to ‘a lot of difficulty’ or ‘cannot do it at all’ to the questions for a domain, then a child was considered to have functional difficulty in that domain [17]. For ‘anxiety’ and ‘depression’, the response category ‘daily’ was considered a functional difficulty [16]. A 5-17 years child was considered to have cognitive challenges if s/he had difficulty in at least one of the domains: communication, learning, remembering and concentrating. The resulting binary variable was labeled as CLRC, and it was the outcome variable of interest in this study.

### Explanatory variables

The primary exposure variable was functional difficulty in the form of anxiety or depression (AD). If a child had functional difficulty either as anxiety or depression, then AD had value ‘yes’; otherwise it was ‘no’. The controlled variables were sex (‘female’, ‘male’), area of residence (‘rural’, ‘urban’), division (‘Barishal’, ‘Chattogram’, ‘Dhaka’, ‘Khulna’, ‘Mymensingh’, ‘Rajshahi’, ‘Rangpur’, ‘Sylhet’), age (‘5-9’, ‘10-14’, ‘15-17’), mother’s education (‘pre-primary or none’, ‘primary’, ‘secondary’, ‘higher secondary+’), mother’s functional difficulty (‘no’, ‘yes’) and wealth index quintile (‘poorest’, ‘second’, ‘middle’, ‘fourth’, ‘richest’).

To gather data on mothers’ functional difficulties, the 2019 MICS employed an adult functioning module created by the Washington Group on Disability Statistics [18]. The ‘mother’s functional difficulty’ variable refers to the information specifically related to the mother of the child in question, as directly sourced from the MICS 2019 dataset. The wealth index was a composite indicator of a household’s living standard. After the survey, all participating households were grouped into quintiles according to their principal component scores, reflecting their relative wealth [16,19]. In this paper, CLRC was treated as the primary outcome variable, while AD was specified as the main exposure of interest.

It is important to note that the Child Functioning Module captures functional difficulties rather than clinical diagnoses. Although difficulties in learning, remembering, or concentrating may overlap with symptoms of anxiety, depression, or other neurodevelopmental conditions such as attention deficit hyperactivity disorder (ADHD), the module assesses these domains separately based on reported functional limitations. As such, the present analysis focuses on the co-occurrence and association of functional difficulties across domains, rather than on differentiating specific clinical disorders.

### Statistical analysis

We performed both bivariate and multivariable analyses on the data. In the bivariate analysis, we calculated the percentage of responses for each category of the covariates and tested for associations between each covariate and the outcome variable using chi-square tests, reporting the corresponding *p*-values [20]. For the multivariable analysis, we applied a random intercept binary logistic regression model to account for potential clustering in the data and to assess the association of covariates while controlling for group-level variability.

A random intercept binary logistic regression model is a powerful extension of the traditional binary logistic regression that accounts for grouped or clustered data [21]. Individuals are nested within districts in MICS data. These clusters can introduce intra-group correlation, where individuals within the same group tend to be more similar to each other than to individuals in other groups, violating the assumption of independence typically required in standard logistic regression. The general form of the random intercept binary logistic regression model can be written as


$$\text{logit}\left\{P(Y_{ij}=1)\right\}=\beta_{0}+\beta_{1}X_{1ij}+\beta_{2}X_{2ij}+\ldots +\beta_{k}X_{kij}+u_{j},$$

where *Y*_*ij*_ is the binary outcome for individual *i* in cluster *j*, $\beta_{0}$ is the overall intercept, representing the average log-odds of success across all clusters, $X_{1ij},X_{2ij},\ldots ,X_{kij}$ are the fixed effect predictors for individual *i* in cluster *j*, each associated with its coefficient $\beta_{1},\beta_{2},\ldots ,\beta_{k}$. These fixed effects are assumed to be constant across all clusters, and *u*_*j*_ is the random intercept specific to cluster *j*, representing the deviation of cluster *j*’s intercept from the overall average intercept. The random effects are assumed to follow a normal distribution with mean 0 and variance $\sigma_{u}^{2}$.

A large $\sigma_{u}^{2}$ suggests substantial differences in the baseline probabilities between clusters, while a small $\sigma_{u}^{2}$ indicates that most clusters have similar baseline probabilities. An important output of the model is the intraclass correlation coefficient (ICC), which measures the proportion of the total variance in the outcome that is attributable to differences between clusters


$$\rho =\frac{\sigma_{u}^{2}}{\sigma_{u}^{2}+\frac{\pi^{2}}{3}}.$$

Here, $\frac{\pi^{2}}{3}$ is the variance of the standard logistic distribution, and the ICC quantifies how much of the variability in the outcome is due to the clustering structure. A higher ICC means that clustering plays a significant role in explaining the outcome. Because the MICS 2019 sample was not self-weighted, we applied the available sampling weights to ensure that the computed statistics accurately represent the population. All statistical analyses were conducted using Stata [22].

## 3 Results

### Bivariate analysis

Table 1 provides a detailed summary of the percentage of children aged 5–17 years in Bangladesh with functional difficulties across various domains in 2019. These domains include communication, learning, remembering, concentrating, anxiety, depression, and two composite measures: CLRC (any difficulty in the form of communication, learning, remembering or concentrating) and AD (any difficulty in anxiety or depression). Overall, the percentages of children experiencing functional difficulties in each domain were as follows: 0.6% in communication, 1.6% in learning, 1.7% in remembering, 0.8% in concentrating, 3.2% in anxiety, 3.7% in depression, 2.3% in CLRC, and 4.3% in AD.

**Table 1:** Percentage of children aged 5-17 years with functional difficulties by domain in Bangladesh, 2019.

Characteristic	Communication	Learning	Remembering	Concentrating	Anxiety	Depression	CLRC	AD	*p*-value
Total	0.6	1.6	1.7	0.8	3.2	3.7	2.3	4.3	
Sex									<0.001
Male	0.6	1.7	1.8	0.9	3.2	3.8	2.5	4.4	
Female	0.6	1.4	1.6	0.8	3.2	3.7	2.0	4.3	
Area									<0.001
Urban	0.4	1.0	1.3	0.7	2.5	2.7	1.6	3.2	
Rural	0.6	1.7	1.8	0.9	3.4	4.0	2.4	4.6	
Division									<0.001
Barishal	1.2	7.1	6.3	3.3	4.1	4.3	9.9	6.0	
Chattogram	0.5	0.8	0.9	0.6	7.6	7.6	1.1	8.2	
Dhaka	0.4	0.8	0.9	0.6	1.8	2.1	1.1	2.5	
Khulna	0.7	1.2	1.7	1.0	0.6	0.8	2.0	1.1	
Mymensingh	0.5	4.6	5.2	1.0	5.0	6.5	6.4	7.8	
Rajshahi	1.0	1.6	1.8	1.1	3.0	5.7	2.5	6.7	
Rangpur	0.5	1.0	1.0	0.8	0.6	0.7	1.2	0.8	
Sylhet	0.5	0.8	0.5	0.3	0.2	0.2	1.1	0.3	
Age									<0.001
5-9	0.7	1.4	1.7	0.9	3.1	4.2	2.3	4.8	
10-14	0.5	2.0	2.0	1.0	3.2	3.6	2.6	4.2	
15-17	0.6	1.1	1.2	0.7	3.3	3.0	1.7	3.8	
Mother’s education									<0.001
Pre-primary or none	0.5	2.2	2.2	0.9	3.6	3.8	2.9	4.5	
Primary	0.6	1.7	2.0	0.9	2.7	3.6	2.5	4.1	
Secondary	0.7	1.2	1.4	1.0	3.4	3.9	1.8	4.5	
Higher secondary+	0.5	0.6	0.5	0.4	2.7	2.9	1.1	3.7	
Mother’s fun. diff.									<0.001
Yes	1.1	7.2	7.0	2.9	6.8	7.6	10.4	9.1	
No	0.6	1.4	1.4	0.8	3.0	3.7	1.9	4.2	
Wealth index									<0.001
Poorest	0.7	2.5	2.7	1.3	2.7	3.6	3.7	4.2	
Second	0.8	2.2	2.4	1.2	3.4	4.3	3.1	4.9	
Middle	0.5	1.3	1.4	0.7	3.4	3.8	1.8	4.5	
Fourth	0.7	1.2	1.2	0.7	3.4	3.4	1.6	4.2	
Richest	0.3	0.4	0.6	0.4	3.0	3.4	0.8	3.8	

The *p*-values were obtained from chi-square tests examining the association between each covariate and CLRC.

Males and females showed similar rates of functional difficulties across most domains. Males had slightly higher percentages in learning (1.7% vs. 1.4%), remembering (1.8% vs. 1.6%), and CLRC (2.5% vs. 2.0%). Both sexes had identical percentages in communication and anxiety. Urban children reported fewer functional difficulties in all domains compared to rural children. For example, anxiety affected 2.5% of urban children versus 3.4% of rural children, while depression affected 2.7% of urban children and 4.0% of rural children. There were substantial regional differences. For instance, Barishal had the highest percentage of children with learning difficulties (7.1%) and communication difficulties (1.2%). Mymensingh showed high percentages across multiple domains: learning (4.6%), remembering (5.2%), and anxiety (5.0%). In contrast, Sylhet recorded the lowest levels of functional difficulty across most domains (e.g., anxiety 0.2%, depression 0.2%, and CLRC 1.1%).

Younger children aged 5-9 years showed a higher prevalence of communication difficulties (0.7%) and AD challenges (4.8%) compared to older age groups. Children aged 10–14 years reported higher percentages in learning (2.0%) and remembering (2.0%) compared to other age groups. Children whose mothers had little or no education showed higher levels of functional difficulties in nearly all domains. For example, children of mothers with pre-primary or no education had higher rates of learning difficulties (2.2%) and CLRC (2.9%) than those whose mothers have higher secondary education or more. There was a clear association between the mother’s functional difficulty status and the child’s likelihood of having a functional difficulty. Children of mothers with functional difficulties showed substantial higher percentages across all domains, with CLRC at 10.4% and AD at 9.1%.

Children from wealthier families generally had fewer functional difficulties. The poorest children had the highest percentages across several domains, including learning (2.5%), remembering (2.7%), and CLRC (3.7%). In contrast, children from the richest households reported the lowest levels of functional difficulties, with only 0.4% experiencing learning difficulties and 0.8% facing CLRC challenges. The table also includes the *p*-values from chi-square tests that examined the association between each covariate and CLRC. The *p*-values indicate that all characteristics (sex, area, division, age, mother’s education, mother’s functional difficulty, and wealth index) had statistically significant associations with CLRC, with all *p*-values being less than 0.001.

### Multivariable analysis

Table 2 presents the results of the multivariable random-intercept logistic regression examining the association between AD and CLRC, after adjusting for relevant child-, maternal-, and household-level covariates. The table includes the odds ratios (OR), *p*-values, and the 95% confidence intervals (CI) for the odds ratios, indicating the likelihood of functional difficulty based on different covariates. Children with functional difficulty in AD have significantly higher odds (OR = 7.82) of having CLRC overall compared to those without, with a *p*-value of <0.001. The 95% CI for the odds ratio is 6.29 to 9.73, indicating a strong and statistically significant association.

**Table 2:** Logistic regression analysis to assess the association between CLRC and AD, controlling other covariates, among children aged 5-17 years in Bangladesh, 2019.

Characteristic	Odds ratio	*p*-value	95% CI for odds ratio
AD (Ref: No)			
Yes	7.82	<0.001	6.29, 9.73
Sex (Ref: Female)			
Male	1.30	0.001	1.12, 1.51
Area (Ref: Rural)			
Urban	1.11	0.375	0.88, 1.39
Division (Ref: Barishal)			
Chattogram	0.17	<0.001	0.08, 0.35
Dhaka	0.23	<0.001	0.12, 0.46
Khulna	0.36	0.005	0.18, 0.73
Mymensingh	0.73	0.476	0.31, 1.73
Rajshahi	0.38	0.010	0.18, 0.80
Rangpur	0.22	<0.001	0.10, 0.47
Sylhet	0.19	0.001	0.07, 0.48
Age (Ref: 5-9)			
10-14	0.94	0.480	0.80, 1.11
15-17	0.65	<0.001	0.51, 0.82
Mother’s education (Ref: Pre-primary or none)			
Primary	0.92	0.447	0.76, 1.13
Secondary	0.80	0.035	0.65, 0.98
Higher secondary+	0.73	0.117	0.49, 1.08
Mother’s functional difficulty (Ref: No)			
Yes	3.00	<0.001	2.37, 3.79
Wealth index quintile (Ref: Poorest)			
Second	0.83	0.059	0.68, 1.01
Middle	0.61	<0.001	0.49, 0.76
Fourth	0.56	<0.001	0.43, 0.73
Richest	0.34	<0.001	0.23, 0.49
Constant	0.06	<0.001	0.03, 0.11

Males are 1.30 times more likely to have CLRC compared to girls (95% CI: 1.12-1.51) indicating a statistically significant difference. Children living in urban areas have slightly higher odds (OR = 1.11) of CLRC compared to those in rural areas, but this association is not statistically significant (*p*-value=0.375). Children from several divisions have significantly lower odds of CLRC compared to those from Barishal. These include Chattogram, Dhaka, Khulna, Rajshahi, Rangpur and Sylhet. Mymensingh does not show a statistically significant difference compared to Barishal (OR = 0.73; 95% CI: 0.31-1.73). Although children aged 10-14 years show no statistically significant difference in CLRC compared to those aged 5-9 years, children aged 15-17 years have significantly lower odds of CLRC (OR = 0.65; 95% CI: 0.51-0.82).

Children whose mothers have secondary education are less likely to have CLRC compared to those whose mothers have pre-primary or no education (OR = 0.80; 95% CI: 0.65-0.98). There is no statistically significant difference for children whose mothers have primary or higher secondary education compared to the reference category (*p* = 0.447 and *p* = 0.117, respectively). Children whose mothers have functional difficulties have significantly higher odds of experiencing CLRC themselves (OR = 3.00; 95% CI: 2.37-3.79). Children from wealthier households tend to have lower odds of functional difficulty. The association for the second quintile is marginally insignificant (OR=0.83; 95% CI: 0.68-1.01).

The logistic regression results reveal that AD plays a substantial role in determining the odds of a child experiencing CLRC. Several other factors significantly contribute to the likelihood of CLRC in children and these include sex, division, age, mother’s functional difficulty, and wealth index. Differences across divisions indicate geographic disparities in CLRC.

The ICC was obtained as 0.1095, meaning that approximately 10.95% of the total variability in the binary outcome was due to differences between clusters. The remaining 89.05% of the variability was attributable to differences within clusters, i.e., individual-level differences. This suggests that there was moderate clustering in the data, meaning that children within the same cluster tend to be more similar in terms of their likelihood of having CLRC compared to children from different clusters.

## 4 Discussion

This study demonstrates a strong association between AD and cognitive challenges related to CLRC among children aged 5-17 years in Bangladesh. Children with functional difficulty in AD are more than seven times as likely to experience CLRC, highlighting the close interrelation between different dimensions of functional difficulties. This robust association, with a highly significant *p*-value of less than 0.001 and a narrow confidence interval, highlights the need to address AD-related difficulties to potentially reduce the prevalence of CLRC.

This finding of the study is in line with previous research cited in the Introduction, which has shown that anxiety and depression in children are associated with impairments in attention, memory, and learning. For example, studies such as Luby et al. [3] and Kaufman et al. [4] reported that depressive symptoms are linked to persistent difficulties in concentration and cognitive performance, while work by Eysenck et al. [6] and Alloway et al. [8] demonstrated that anxiety-related disruptions in attentional control and working memory adversely affect learning outcomes. Our results extend these findings by demonstrating a similar pattern of association at the population level in a low- and middle-income country context using a standardized functional disability framework.

The analysis also reveals significant gender disparities in functional difficulty, with males being 30% more likely to experience CLRC compared to females. While this difference is statistically significant, it raises important questions about the underlying social, biological, or environmental factors that may contribute to the increased vulnerability of boys to such difficulties. Geographic disparities were evident in the data, with children from several divisions-including Chattogram, Dhaka, Khulna, Rajshahi, Rangpur, and Sylhet-having significantly lower odds of CLRC compared to those from Barishal. These findings suggest the potential influence of regional factors such as healthcare access, socioeconomic conditions, and cultural practices in shaping the prevalence of cognitive difficulties in children. Mymensingh, however, did not show a statistically significant difference from Barishal, indicating that further research may be needed to explore the specific conditions within these divisions that contribute to these variations.

Age also plays an important role in CLRC outcomes. While children aged 10-14 do not exhibit a significant difference from the 5-9 age group, older children (15-17 years) has significantly lower odds of experiencing CLRC. This finding could reflect the developmental nature of certain functional difficulties, where older children may have developed coping mechanisms or received interventions that mitigate the impact of earlier difficulties. The education level of the mother emerges as a protective factor, with children whose mothers has secondary education showing lower odds of CLRC compared to those whose mothers has no or pre-primary education. However, this protective effect do not extend significantly to children whose mothers has primary or higher secondary education, suggesting that the benefits of maternal education may be more nuanced.

The strong association between a mother’s functional difficulty and her child’s likelihood of experiencing CLRC (OR = 3.00) highlights the inter-generational transmission of health challenges, indicating that interventions targeting maternal health and functional capacity could have a positive ripple effect on child outcomes. Wealthier households are also associated with lower odds of CLRC, although the effect for the second wealth quintile is marginally insignificant. This aligns with broader global evidence linking socio-economic status with better health outcomes, emphasizing the need for targeted interventions to support children from lower-income households who may be at higher risk of functional difficulties.

Maternal education and household wealth are closely interconnected dimensions of socioeconomic status, and their associations with cognitive challenges in children should be interpreted jointly. Higher maternal education may enhance cognitive stimulation, health-related knowledge, and caregiving practices within the household, while greater household wealth can improve access to nutrition, healthcare, and educational resources. Although these factors are associated, their simultaneous inclusion in the model suggests that each captures a distinct pathway through which socioeconomic advantage may reduce the likelihood of CLRC. Maternal education and household wealth may work together, with education supporting effective caregiving and wealth enabling access to resources, jointly influencing children’s cognitive functioning.

Overall, this study highlights the many-sided nature of functional difficulties in children, with significant contributions from individual, familial, geographic, and socioeconomic factors. The strong association between AD and CLRC, combined with regional, gender, and socioeconomic disparities, highlights the need for comprehensive interventions that address these multiple dimensions to reduce the burden of childhood functional difficulties in Bangladesh.

## 5 Strengths and limitations

This study utilized data from a large, nationally representative sample of children aged 5-17 years in Bangladesh, making the findings generalizable to the broader population. The inclusion of children across diverse geographic regions allows for an in-depth exploration of regional disparities in functional difficulties.

The study’s cross-sectional nature limits the ability to draw causal inferences. While associations between covariates and CLRC are identified, it is not possible to determine whether these factors directly cause functional difficulties or if there are other unmeasured confounders. The use of self-reported or caregiver-reported data for functional difficulties could introduce measurement error or bias. Caregivers may under-report or overestimate the severity of a child’s difficulties, potentially affecting the accuracy of the associations observed. Some important factors that could influence child functional difficulties, such as access to healthcare services and environmental conditions were not included in the analysis. These unmeasured confounders could potentially bias the observed associations.

In summary, while the study offers valuable insights into the association between AD and CLRC difficulties of children in Bangladesh, it is important to interpret the findings in light of these limitations, particularly the cross-sectional design and potential unmeasured confounders.

## 6 Conclusion

This study establishes meaningful evidence on the association between AD and CLRC-related functional difficulties in children. Significant disparities in the prevalence of CLRC were observed based on gender, age, geographic region, maternal education, wealth, and maternal functional difficulties. These findings highlight the complex interplay of individual, familial, and socio-economic factors in shaping child health outcomes.

The results suggest that addressing functional difficulties requires targeted interventions, particularly focusing on vulnerable groups such as children with AD difficulties, boys, children from lower-income households, and those whose mothers have disabilities. The regional variations point to potential geographic inequities in access to healthcare or other resources, suggesting the need for region-specific policies to reduce these disparities.

While the study contributes to the understanding of childhood functional difficulties in a low-resource setting, further longitudinal research is needed to explore causal relationships and identify long-term solutions. Addressing functional difficulties in childhood can have far-reaching implications for children’s development and well-being, making it a critical area for policy and intervention efforts.
